# RASGRP2在肺腺癌中的表达及对免疫微环境的影响

**DOI:** 10.3779/j.issn.1009-3419.2021.105.01

**Published:** 2021-06-20

**Authors:** 世康 赵, 鑫 金, 嵩 徐

**Affiliations:** 300052 天津，天津医科大学总医院肺部肿瘤外科；天津市肺癌研究所，天津市肺癌转移与肿瘤微环境实验室 Department of Lung Cancer Surgery; Tianjin Key Laboratory of Lung Cancer Metastasis and Tumor Microenvironment, Tianjin Lung Cancer Institute, Tianjin Medical University General Hospital, Tianjin 300052, China

**Keywords:** 肺腺癌, 免疫治疗, RASGRP2, 预后, 免疫微环境, Lung adenocarcinoma, Immunotherapy, RASGRP2, Prognosis, Immune microenvironment

## Abstract

**背景与目的:**

肺癌是全球发病率和死亡率的居高不下的恶性肿瘤。近年来，随着新型药物的出现和治疗模式的优化，特别是免疫治疗在临床的应用，肺癌患者的预后已经有了一定的改善。但免疫治疗获益的患者仍然有限，因此我们想要寻找新的生物标志物用于预测肺腺癌患者的预后并探索其对免疫微环境的影响。

**方法:**

使用癌症基因组图谱（The Cancer Genome Atlas, TCGA）数据库下载肺腺癌患者的测序结果及临床资料。利用人类蛋白质图谱数据库明确RASGRP2在肺腺癌中分布情况，使用*Kaplan-Meier* Plotter数据库探索RASGRP2表达情况与肺腺癌患者预后之间的联系。对RASGRP2高低表达患者进行KEGG及GO基因富集分析。使用TCGA数据库分析RASGRP2共表达基因并使用TIMER数据库计算RASGRP2及其共表达基因的免疫相关淋巴浸润情况。最后使用TIMER 2.0数据库分析RASGRP2表达量与免疫检查点表达的联系。

**结果:**

RASGRP2在肺腺癌中低表达，与患者预后密切相关。RASGRP2高表达参与造血细胞形成及细胞黏附，且在T细胞活化进程中有着重要作用。通过TCGA数据库分析发现ZAP70、TBC1D10C、RASAL3、FGD2、CD37、ACAP1与RASGRP2显著相关。以上基因高表达会引起CD8^+^ T细胞、记忆CD4^+^ T细胞比例升高以及中性粒细胞和Treg细胞比例降低。最后，我们发现RASGRP2表达量与常见免疫检查点CD274、CTLA4、LAG3、TIGIT等表达量显著相关。

**结论:**

RASGRP2在肺腺癌中异常表达且影响免疫相关细胞浸润水平，可能会影响免疫治疗疗效。

## Abstract (en)

**Background and objective:**

Lung cancer still has the highest incidence rate and mortality rate nowadays. In recent years, with the emergence of new drugs and the optimization of treatment mode, especially the clinical application of immunotherapy, the prognosis of lung cancer patients has been improved. However, the benefits of immunotherapy are still limited. Therefore, it is necessary to find new biomarkers to predict the prognosis of lung adenocarcinoma patients and explore its impact on the immune microenvironment.

**Methods:**

The Cancer Genome Atlas (TCGA) database was used to analyze the gene sequencing and clinical data of patients with lung adenocarcinoma. The distribution of RASGRP2 in lung adenocarcinoma was determined by using the human protein mapping database. The *Kaplan-Meier* plotter database was used to explore the relationship between the expression of RASGRP2 and the prognosis of patients with lung adenocarcinoma. KEGG and GO gene enrichment analysis was performed in patients with high and low expression of RASGRP2. TCGA database was used to analyze the co-expression genes of RASGRP2 and TIMER database was used to calculate the immune related lymphoid infiltration of RASGRP2 and its coexpression genes. The relationship between RASGRP2 expression and immune checkpoint expression was analyzed by using TIMER 2.0 database.

**Results:**

We found that RASGRP2 was low expressed in lung adenocarcinoma, and its expression level was related to the prognosis of patients. The high expression of RASGRP2 was involved in the process of hematopoietic cell formation and cell adhesion, and RASGRP2 played an important role in the process of T cell activation. Through TCGA database analysis, ZAP70, TBC1D10C, RASAL3, FGD2, CD37 and ACAP1 were significantly correlated with RASGRP2. The high expression of these genes leaded to the increase of the proportion of CD8^+^ T cells, memory CD4^+^ T cells, and the decrease of the proportion of neutrophils and Treg cells. Finally, we found that the expression of RASGRP2 was significantly correlated with the expression of CD274, CTLA4, LAG3 and TIGIT.

**Conclusion:**

RASGRP2 was abnormally expressed in lung adenocarcinoma and correlated with the infiltration level of immune related cells, which might influence the efficacy of immunotherapy.

肺癌是现今全球发病率第二及致死率最高的恶性肿瘤^[1]^。据中国癌症统计2015年数据^[2]^显示，我国因癌症死亡的病人数目已达281.4万人，其中肺癌是导致发病率和死亡率最高的癌种。肺癌根据病理组织类型主要被分为小细胞肺癌（small cell lung cancer, SCLC）和非小细胞肺癌（non-small cell lung cancer, NSCLC）。其中腺癌是NSCLC中最常见的类型^[3]^。近年来随着靶向治疗及免疫治疗的兴起，肺癌死亡率较前有所下降，但免疫治疗在癌症患者中有效率仅为20%-30%。现已有多种针对免疫治疗疗效预测的生物标志物，例如程序性死亡受体配体1（programmed cell death ligand 1, PD-L1）、肿瘤突变负荷（tumor mutation burden, TMB）、微卫星不稳定（microsatellite instability, MSI）等，但其灵敏性及特异度均差强人意^[4]^，因此迫切需要找到更好的免疫治疗生物标志物。

Ras蛋白特异鸟嘌呤核苷酸释放因子2（RASGRP2）是Ras蛋白鸟氨酸释放蛋白家族（RASGRP）中的一员，可以激活小GTPases蛋白中Ras家族中的Rap1^[5]^。Wu等^[6]^研究结果提示RASGRP2可以有效预测结肠癌患者的预后，此外，Essa等^[7]^发现RASGRP2表达量与肝癌患者总生存期相关。然而针对RASGRP2在肺腺癌患者中的表达情况及其对免疫微环境影响均未有报道。在本项研究中，我们通过收集癌症基因组图谱（The Cancer Genome Atlas, TCGA）的肺癌测序结果及临床数据，在分析RASGRP2在肺腺癌患者人群中表达情况的基础上，又通过多项数据库分析其表达量对免疫微环境的影响，帮助我们确定可以预测肺癌预后的潜在生物标志物，并希望有助于筛选更适合免疫治疗获益的患者人群。

## 1 材料与方法

### 1.1 TCGA数据库检索

研究主要通过检索TCGA数据库（https://www.cancer.gov/tcga）搜集数据^[8]^。TCGA包括RNA测序、miRNA测序、DNA测序等多种测序结果以及相关患者的临床信息。我们从TCGA数据库下载了所有LUAD患者的转录组和临床数据，整理得到523例患者的转录组信息及临床数据。

### 1.2 人类蛋白质图谱

人类蛋白质图谱项目（Human Protein Atlas, HPA）（https://www.proteinatlas.org/）致力于研究人类蛋白质的组织和细胞分布信息，我们通过该数据库中病理图谱来探讨RASGRP2在肺腺癌中分布情况。

### 1.3 计算免疫相关淋巴细胞浸润情况

使用TIMER（https://cistrome.shinyapps.io/timer/）和TIMER 2.0（http://timer.cistrome.org/）数据库计算出RASGRP2表达量对B细胞、T细胞、中性粒细胞、单核细胞、巨噬细胞等免疫细胞浸润情况的影响。

### 1.4 功能富集分析

通过RASGRP2表达量将TCGA数据库中肺腺癌患者分为高低表达两组，使用R语言“Cluster Profiler”包行后续基因富集分析。

### 1.5 *Kaplan-Meier* Plotter数据库

通过使用*Kaplan-Meier* Plotter数据库（http://kmplot.com/analysis/）^[9]^绘制生存曲线。探索*RASGRP2*的基因与蛋白水平表达对于肺腺癌患者生存期的影响。

### 1.6 统计学分析

使用*Wilcoxon*检验对两组样品进行显著性分析，应用*Pearson*卡方检验分析RASGRP2表达与临床特征变量的相关性。单因素和多因素分析采用*Cox*比例风险回归模型。*P* < 0.05为差异具有统计学意义。

## 2 结果

### 2.1 RASGRP2高低表达患者临床资料分析

本研究通过检索TCGA数据库，共下载了523例肺腺癌患者的转录组信息及临床数据，其中RASGRP2高表达患者259例，RASGRP2低表达患者264例。通过性别、年龄、吸烟情况、肿瘤原发灶-淋巴结-转移（tumor-node-metastasis, TMN）分期对其细分后，我们发现RASGRP2低表达患者中，女性患者比例较高。而RASGRP2表达情况与年龄及吸烟状况之间没有统计学意义。通过TNM分期可以看到，RASGRP2高表达患者分期较早（表 1）。

**表 1:** RASGRP2高低表达肺腺癌患者临床基本信息 Analysis of clinical data of lung adenocarcinoma patients with high and low expression of RASGRP2

Characteristic		Expression of RASGRP2		*P*
		High(*n*=259)	Low(*n*=264)	
Gender	Male	137 (52.9%）	106 (40.2%)	0.003, 8
	Femlae	122 (47.1%）	158 (59.8%)	
Age (yr)	≤60	89 (34.4%)	71 (26.9%)	0.055, 4
	> 60	158 (61.0%)	184 (69.7%）	
Smoking status	Never	31 (12.0%)	44 (16.7%)	0.134, 6
	Ever	220 (84.9%)	212 (80.3%)	
T stage	T1/T2	236 (91.1%)	214 (81.1%)	0.004, 7
	T3/T4	21 (8.1%)	42 (15.9%)	
N stage	N0	181 (69.9%)	154 (58.3%)	0.015, 2
	N1/N2/N3	74 (28.6%)	100 (37.9%)	
M stage	M0	182 (70.3%)	171 (64.8%)	0.204, 8
	M1	16 (6.2%)	8 (3.0%)	
Stage	I/Ⅱ	215 (83%)	190 (72.0%)	0.000, 4
	Ⅲ/Ⅳ	35 (13.5%)	70 (26.5%)	

### 2.2 RASGRP2在肺腺癌中表达情况及预后分析

通过TIMER数据库我们发现RASGRP2在肺腺癌患者的癌旁组织中显著高表达（图 1A），之后为证实其在肺腺癌中低表达，我们使用HPA数据库搜寻RASGRP2在肺腺癌组织中免疫组化染色结果，发现RASGRP2在癌旁组织中相比癌组织显著高表达（图 1B）。进一步通过*Kaplan-Meier* Plotter数据库，我们发现RASGRP2高表达的患者总生存期显著优于低表达患者（图 1C）。以上结果说明RASGRP2在肺腺癌癌旁组织相比癌组织显著高表达，且RASGRP2高表达患者预后更佳。

**图 1:**
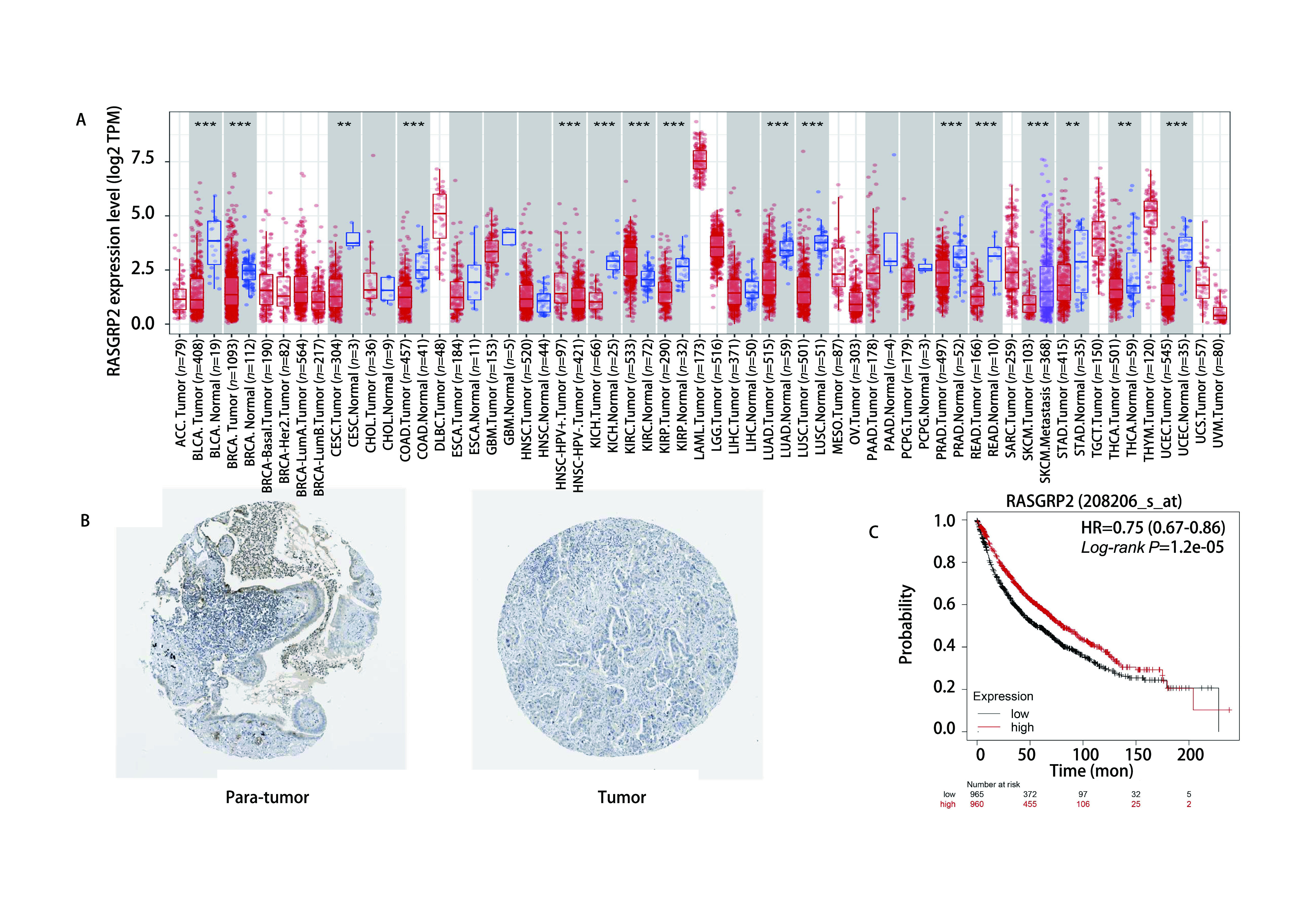
RASGRP2在肺腺癌中表达与预后情况。A：TIMER数据库预测RASGRP2在肺腺癌组织表达情况；B：RASGRP2在肺腺癌及癌旁组织中蛋白表达情况；C：RASGRP2表达与肺腺癌患者预后的关系。 The expression level and prognostic value of RASGRP2 in patients with lung adenocarcinoma. A: Increased or decreased expression of RASGRP2 in cancers compared with adjacent normal tissue in TIMER database; B: RASGRP2 protein expression in normal tissue and tumor tissue of patients with lung adenocarcinoma; C: The relationship between RASGRP2 expression and prognosis of patients with lung adenocarcinoma.

### 2.3 通路富集分析明确RASGRP2调节相关通路

对TCGA数据库中RASGRP2高表达及低表达患者进行基因富集分析后，我们发现共有234个基因上调以及3个基因下调（图 2A）。通过GO及KEGG富集分析后，我们发现其参与造血细胞形成及细胞黏附进程中（图 2B），且在GO富集分析中可以发现RASGRP2在T细胞活化进程中其着重要作用，这提示我们该基因可能可以参与免疫微环境的调节。

**图 2:**
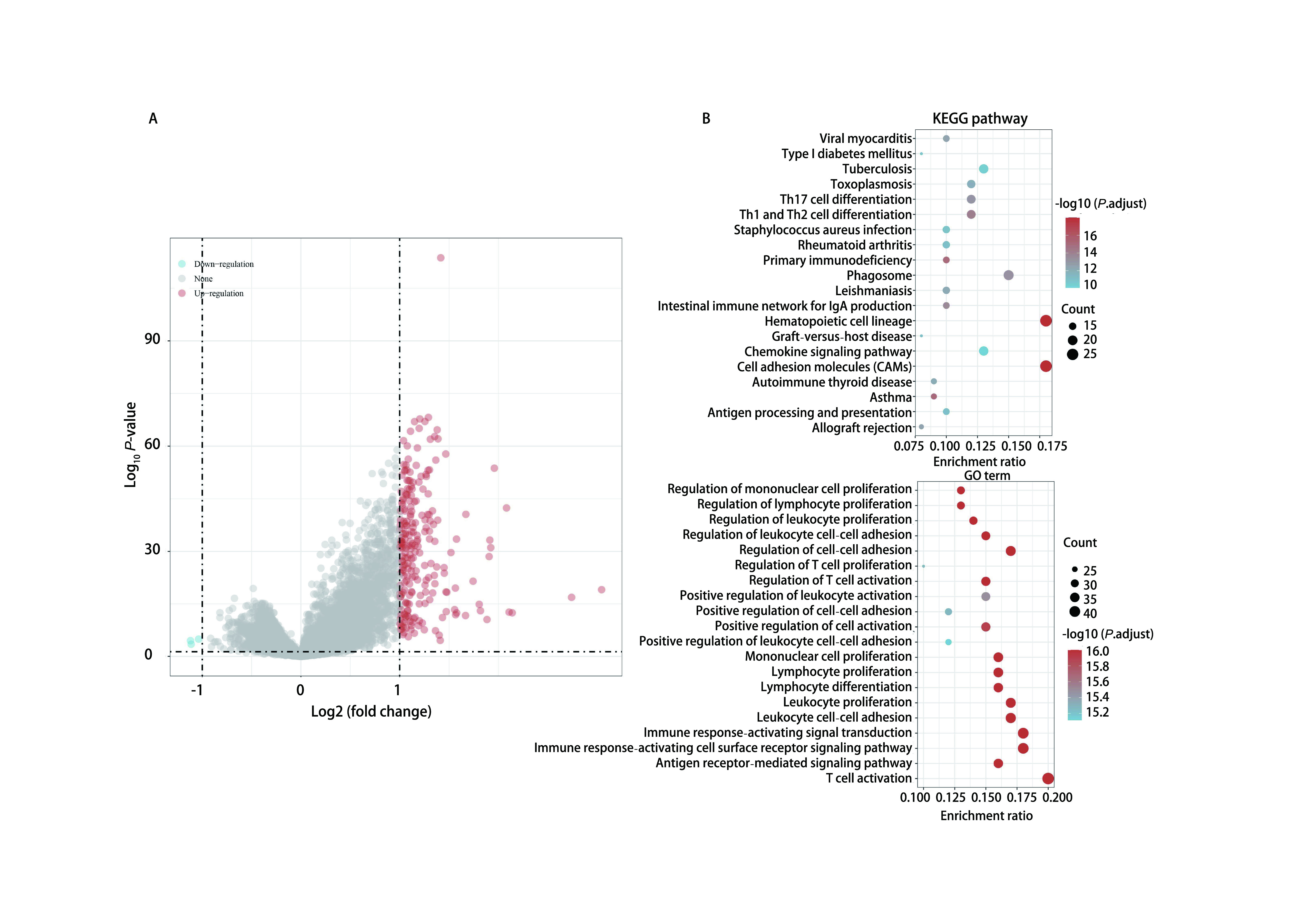
RASGRP2高表达患者基因通路富集分析。A：火山图；B：KEGG及GO富集分析。 GSEA pathways enriched in samples with high RASGRP2 expression. A: Volcanic map; B: KEGG and GO analysis.

### 2.4 探索在肺腺癌中与RASGRP2共表达的基因

在明确RASGRP在肺腺癌中低表达且可能参与免疫调节过程后，我们通过TCGA数据库探索在肺腺癌发生发展过程中，与RASGRP2可能存在共表达的基因。结果发现ZAP70、TBC1D10C、RASAL3、FGD2、CD37、ACAP1与RASGRP2显著相关（图 3A），之后我们又通过TIMER数据库对此结果进行了验证（图 3B）。

**图 3:**
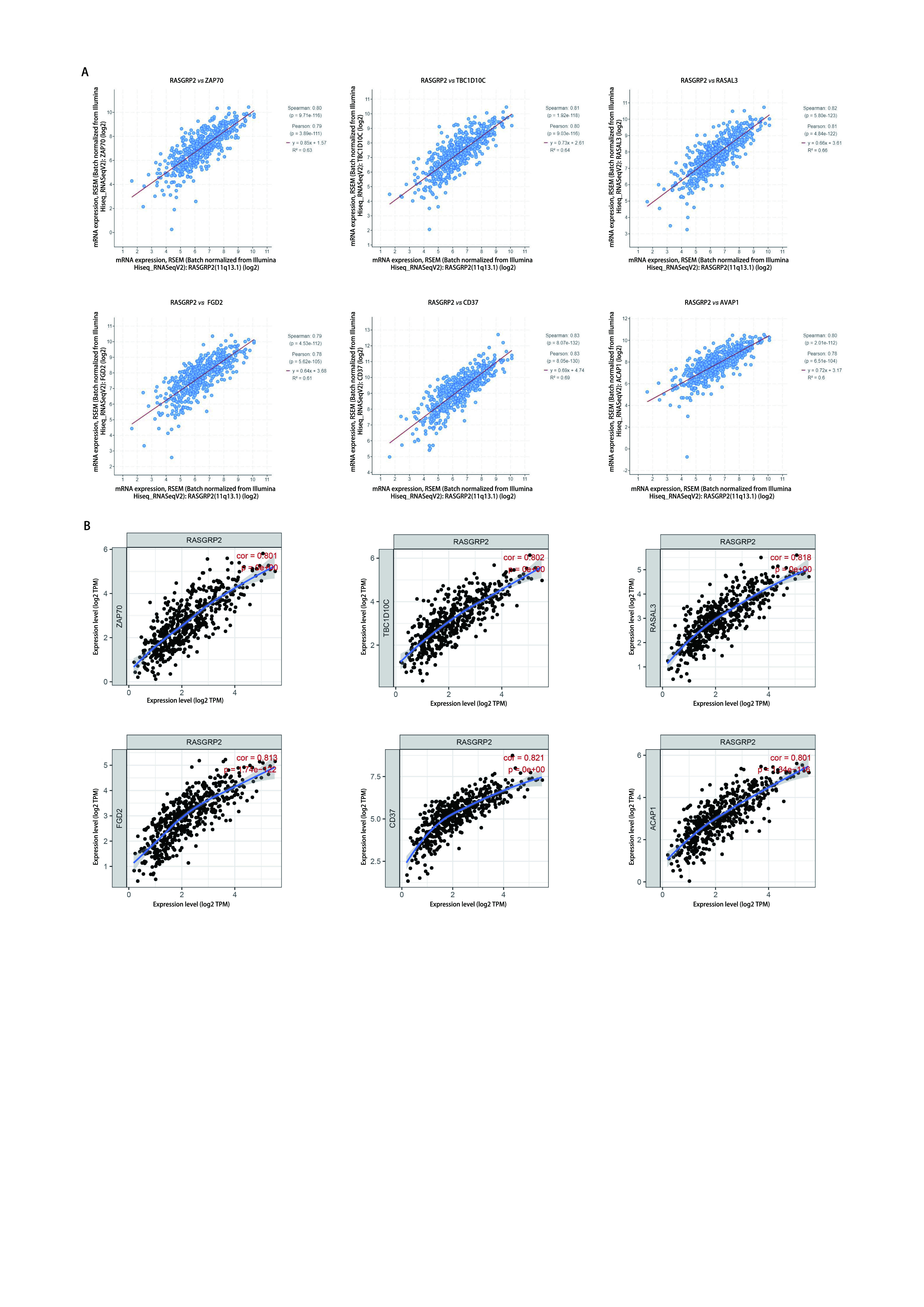
RASGRP2共表达基因分析。使用TCGA（A）及TIMER（B）数据库分析与RASGRP2共表达的基因。 Co-expressed genes of RASGRP2 in lung adenocarcinoma. The genes co-expressed with RASGRP2 in lung adenocarcinoma were assessed in TCGA (A) and TIMER (B) database.

### 2.5 RASGRP2及其共表达基因对肺癌组织免疫相关细胞浸润水平的影响分析

利用TIMER数据库分析RASGRP2对免疫相关细胞浸润水平影响，研究共分析22种免疫细胞，包括B细胞、T细胞、巨噬细胞、单核细胞、中性粒细胞等。结果发现RASGRP2高表达会引起CD8^+^ T细胞、记忆CD4^+^ T细胞比例升高，中性粒细胞、Treg细胞比例降低（图 4A），这说明RASGRP2高表达会促进免疫效应细胞的募集并降低免疫抑制细胞比例，从而对肿瘤起到抑制作用。同时，我们发现RASGRP2的共表达基因也对免疫浸润细胞起到相似的调节作用（图 5）。

**图 4:**
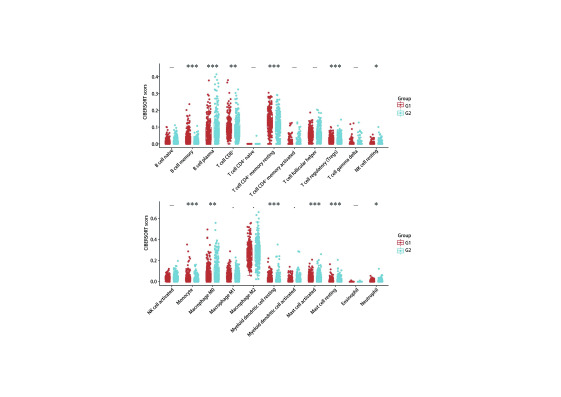
RASGRP2不同表达的肺腺癌患者免疫微环境改变（G1：高表达，G2：低表达） Changes of immune microenvironment in lung adenocarcinoma patients with different expression of RASGRP2 (G1: high expression of RASGRP2, G2: low expression of RASGRP2). *P < 0.05; **P < 0.01; ***P < 0.001. NK: natural killer.

**图 5:**
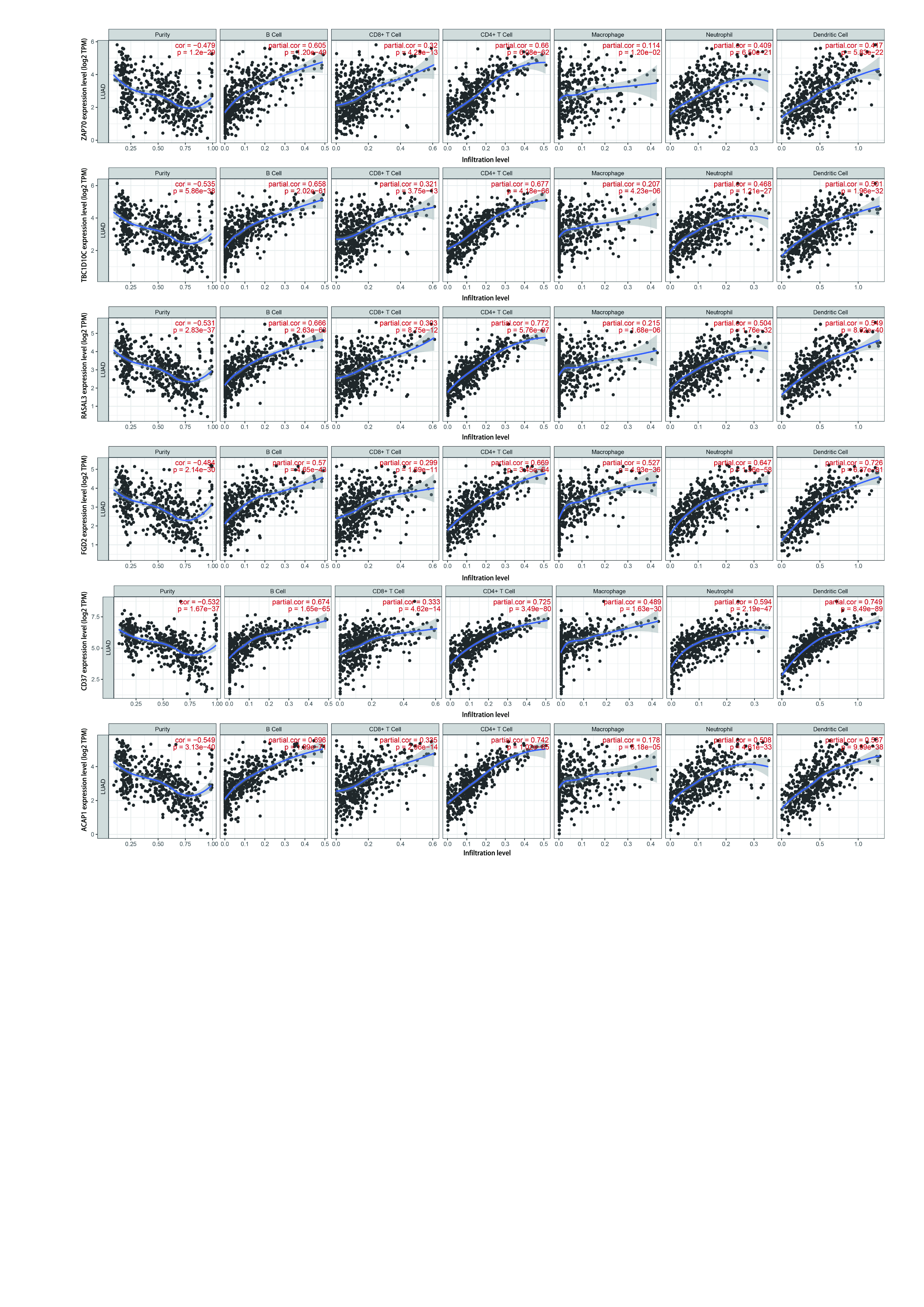
RASGRP2共表达基因对免疫浸润细胞变化水平影响 Relationship between RASGRP2 co-expressed genes and tumor-infiltrating immune cells

### 2.6 RASGRP2表达与免疫检查点密切相关

汇总RASGRP2高低表达患者并将其分别G1（高表达）及G2（低表达）组，使用TIMER 2.0数据库分析发现，RASGRP2表达量与常见免疫检查点CD274、CTLA4、LAG3、TIGIT等表达量显著相关（图 6），提示我们RASGRP2可能与免疫检查点密切相关，且有望在免疫治疗中发挥作用。

**图 6:**
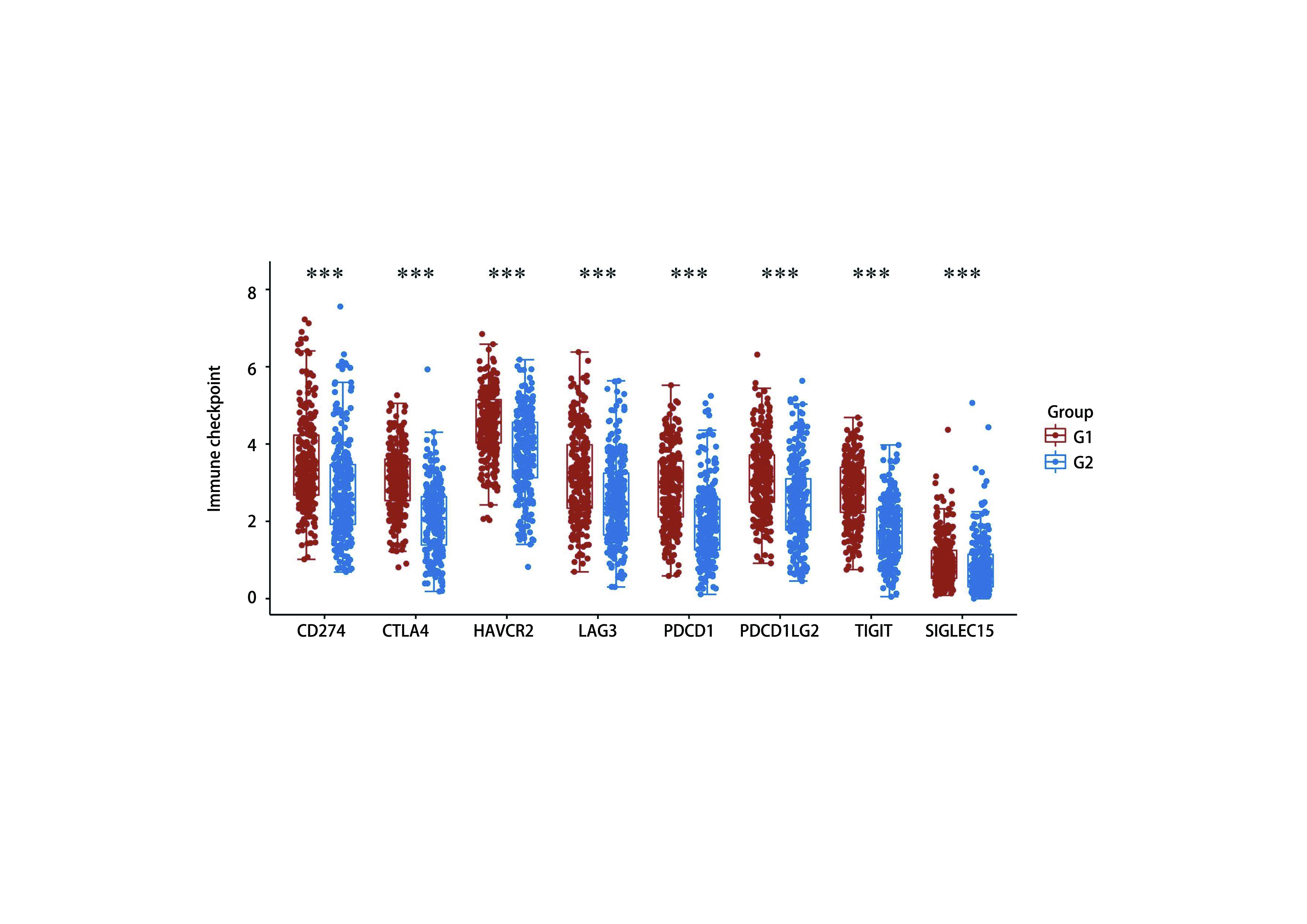
RASGRP2表达量对免疫检查点表达的影响 The relationship between RASGRP2 and immune checkpoints. ***P < 0.001.

## 3 讨论

肺癌作为当今发病率及死亡率极高的恶性肿瘤，其治疗方式也日益增多^[10]^。目前以PD-1/PD-L1为靶点的免疫检查点抑制剂无论在晚期NSCLC的一线和二线治疗，局部晚期NSCLC的巩固治疗，还是早期NSCLC的新辅助治疗均可以为患者带来获益，在NSCLC的综合治疗中显示出重要地位^[11, 12]^。尽管肿瘤免疫治疗在一些患者身上取得了显著且持久的疗效，但抗PD-1/PD-L1治疗的整体响应率只有20%-30%。一方面，预测反应率的生物标志物研究的不足限制了临床患者治疗策略的有效性的提高；另一方面，由于机体免疫调节信号通路错综复杂，耐药成为免疫治疗发展的一大难题和挑战。在本项研究中，我们主要探讨了RASGRP2在肺腺癌中表达情况以及其对免疫浸润细胞的影响。

本研究首先发现RASGRP2在肺腺癌组织中低表达并通过多个数据库对此结果进行验证，通过TCGA数据库患者的临床资料汇总，我们发现RASGRP2与患者性别及分期相关，并且RASGRP2高表达患者的分期较早。在确定RASGRP2在腺癌癌旁组织高表达后，我们发现RASGRP2高表达患者同样具有更好的生存期，以上结果提示*RASGRP2*可能是一个抑癌基因。为了进一步探索RASGRP2在腺癌中调控机制，我们进行了通路富集分析，结果发现RASGRP2高表达与T细胞活化密切相关，这提示我们RASGRP2可能参与了免疫浸润细胞调节过程中。

同时我们使用TCGA数据库基因谱分析发现部分与RASGRP2可能存在共表达的基因，这些基因伴随着RASGRP2在腺癌组织中低表达。在TIMER数据库中，我们探索了RASGRP2表达对于免疫微环境的影响。结果如预期，RASGRP2高表达会引起免疫效应细胞的比例升高，同时我们发现免疫抑制细胞比例的降低。对其共表达基因进行分析后，发现他们伴随着RASGRP2表达改变时也会引起免疫效应细胞如CD4^+^ T细胞、CD8^+^ T细胞比例的升高。免疫微环境中效应细胞比例的升高往往提示患者的较好预后^[13, 14]^。最后，我们通过TIMER 2.0数据库分析免疫检查点的变化情况，结果发现RASGRP2表达与免疫检查点联系紧密，这提示着该基因有望成为用于预测免疫治疗的生物标志物。

综上所述，本研究通过使用多种数据库发现RASGRP2在肺腺癌中的表达情况及对免疫微环境的影响。我们发现RASGRP2在肺腺癌中低表达且与肺腺癌患者预后相关，其可能通过激活效应T细胞从而对肿瘤起到杀伤功能。此外，RASGRP2与免疫检查点密切相关，有望在今后免疫治疗过程中起到指导作用。
